# Supplementary material for: Parasite-Probiotic Interactions in the Gut: Bacillus sp. and Enterococcus faecium Regulate Type-2 Inflammatory Responses and Modify the Gut Microbiota of Pigs During Helminth Infection
Source: Front Immunol. 2022 Jan 5;12:793260. doi: 10.3389/fimmu.2021.793260 (PMC8766631; doi:10.3389/fimmu.2021.793260)
Supplement: Supplementary file 2 [file DataSheet_2.pdf]

Supplementary Material

Parasite-probiotic interactions in the gut: *Bacillus* sp. and *Enterococcus faecium* regulate type-2 inflammatory responses and modify the gut microbiota of pigs during helminth infection

Laura J. Myhill[1]*, Sophie Stolzenbach[1]*, Helena Mejer[1], Lukasz Krych[2], Simon R. Jakobsen[1], Witold Kot[3], Kerstin Skovgaard[4], Nuria Canibe[5], Peter Nejsum[6], Dennis S. Nielsen[2], Stig M. Thamsborg[1]*, Andrew R. Williams[1]*

Contents:

Supplementary Figures 1-3

Supplementary Tables 1-5

A

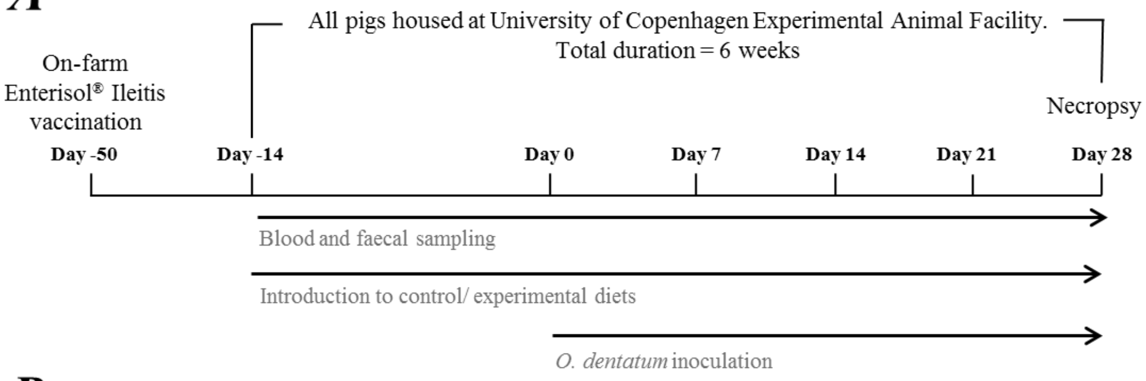

B

	**Standard, non-pelleted feed (‘Control’)**	**Standard feed + Probiotic Mix 1 (‘BBE’)**	**Standard feed + Probiotic Mix 2 (‘LB’)**
**No infection**	n = 8	n = 8	n = 8
***Oesophagostomum dentatum* infection**	n = 8	n = 8	n = 8

Supplementary Figure 1. Experimental set up. (A) At day -14, 48 pigs arrived and were fed one of three diets. At day 0, 24 pigs were inoculated with 25 *O. dentatum* third stage larvae (L3) / kg body weight, followed by similar inoculations three times a week until day 28 post-infection (p.i.). (B) Final number of animals per treatment group at termination of study at day 28 p.i.

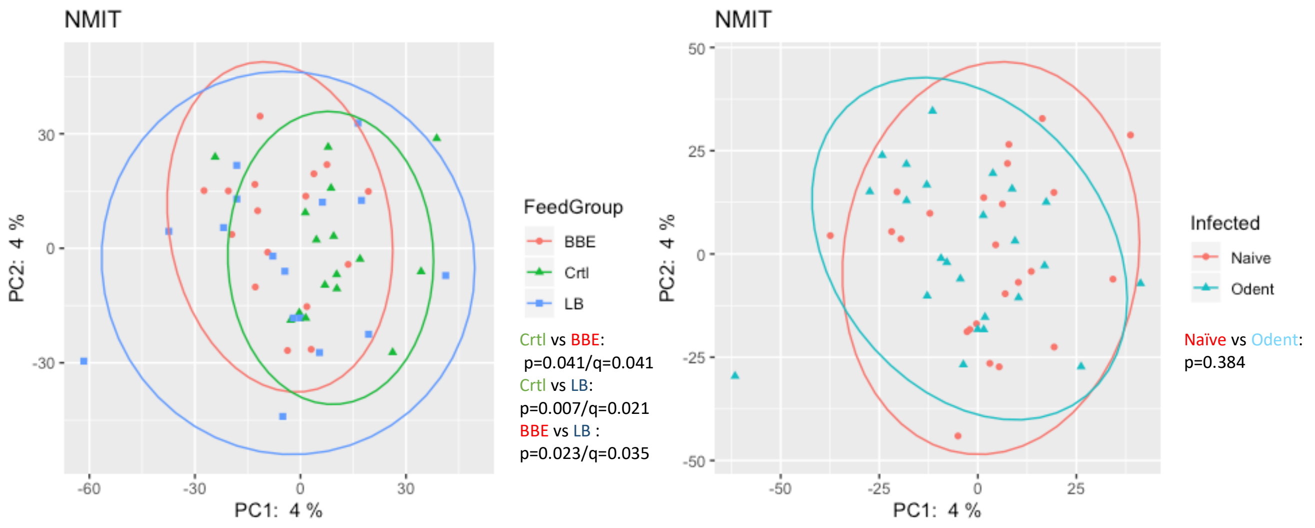

Supplementary Figure 2

Figure S1: Pooled NMIT analysis according to probiotic supplementation (left) or *Oesophagostomum dentatum* (*Odent*) (right).

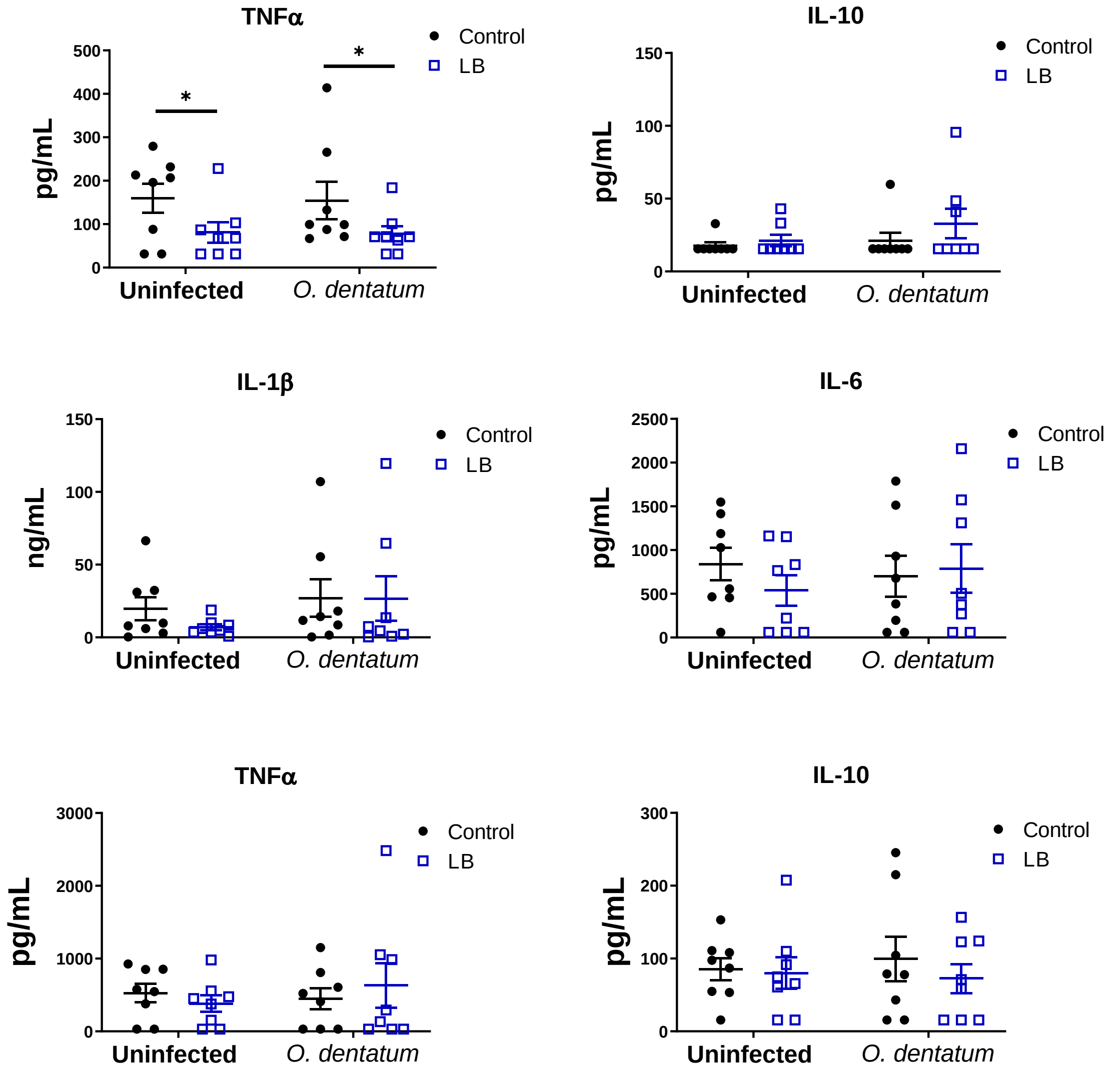

Supplementary Figure 3. ***Ex vivo*** **cytokine secretion in pigs given LGG/Bb12**

A) Phytohaemagglutinin-induced secretion of TNFα and IL-10 in ileal-caecal lymph node cultures. Pigs were either uninfected or infected with *O. dentatum* for 28 days, with or without supplementation of a mixture of LGG and Bb12 (LB). **B)** LPS-induced secretion of IL-1β, IL-6, TNF α and IL-10 in peripheral blood mononuclear cells from pigs infected with *O. dentatum* for 28 days or uninfected pigs, with or without supplementation of LB. * $p < 0.05$ by GLM analysis. n=8 pigs per treatment group.

Supplementary Table 1. Primers used for qPCR.

Gene	Forward Primer (5' – 3')	Reverse Primer (5' – 3')	Amplicon Length
IL1A	TGTGCTAAATAACCTGGATGAGG	GGTTCGTCTTCGTTTTGAGC	135
IL1B	CCAAAGAGGGACATGGAGAA	GGGCTTTTGTTCTGCTTGAG	123
IL8	GAAGAGAACTGAGAAGCAACAACA	TTGTGTTGGCATCTTTACTGAGA	99
IL12B	GACCAGAAAGAGCCCAAAAAC	AGGTGAAACGTCCGGAGTAA	70
IL15	CGTCATTTTGCAAGAGTCCA	TGGACGATAAACTGCTGTTTGC	86
IL17A	GAGGTACCCCTCCGTGATCT	CTTCCTTCCCTTCAGCATTG	71
IFNG	:CCATTCAAAGGAGCATGGAT	TTCAGTTTCCCAGAGCTACCA	76
TNF	CCCCCAGAAGGAAGAGTTTC	CGGGCTTATCTGAGGTTTGA	92
TLR2	CGGAGGTTGCATATTCCACAG	TGTGAAAGGGAACAGGGAAC	128
TLR3	ATTGTGCAAAAGATTCAAGGTG	TCTTCGCAAACAGAGTGCAT	130
TLR4	TGGTGTCCCAGCACTTCATA	CAACTTCTGCAGGACGATGA	116
TLR7	AGAAGCCCCTTCAGAAGTCC	GGTGAGCCTGTGGATTTGTT	93
CD40	TGAGAGCCCTGGTGGTTATC	GCTCCTTGGTCACCTTTCTG	90
CD86	CATCGTCTGTGTCCTGCAAC	CACAGGTGGCTTTGCATCTA	82
CD163	CACATGTGCCAACAAAATAAGAC	CACCACCTGAGCATCTTCAA	130
PRF1	CTATGGCTGGGACGATGACC	CATGGTTCAAGGCGCACATC	86
GZMA	AAGGGGATCTTCAGCTGCTT	GGGGTTCGACATCTTTTCCT	99
GZMB	CCAGGACCAGGATAATCGAA	GGGTGACGTTGATTGAGCTT	101
KLRK	GATGGTTCCATCCTCTCACC	TGAGCCATAGACTGCACAGC	75
INOS	CAGCCCAAGGTCTATGTTCAAG	ATAGAGGTGGCCTTGCTCCT	90
CCL3	CTCTGCAGCCAGGTCTTCTC	CTACGAATTTGCGAGGAAGC	97
CXCL9	AGCAGTGTTGCCTTGCTTTT	ATGCAGGAACAACGTCCATT	92
IL4	GCAAACATGACCTGTTCTGTG	GCTTCAACACTTTGAGTATTTCTCC	105
IL5	GGGGAAAGATGGAGAGTAACG	CTTTCCATTGTCCACTCGGTA	83
IL13	CCAAGCGAGCAAGTTCCTG	AACTACCCGTGGCGAAAAAT	110
ARG1	TCCAAGGTCTGTGGGAAAAG	ATCGCCATACTGTGGTCTCC	108
CCL17	GGGTGGTACCAGACCTCAGA	GTCCTTGGGGTCAGAACAGA	90
CCL22	CCCTGCGTGTGGTGAAGTAT	ATCTCTCGGTCCCTCAAGGT	88
CCL26	CTGCTTCCAATACAGCCACA	AGCAGCTGTTCCTGGTGAAT	74
CCR4	GGACCCCTTACAATGTGGTG	GAATGGCGTAGTCCAGGTGT	96
IL10	TACAACAGGGGCTTGCTCTT	GCCAGGAAGATCAGGCAATA	110
TGFB1	TCACCGGGGCTGTATTTAAG	AAGGAAGACCCCAGTCAGGT	110
FOXP3	GAAGGACAGCACCCTTTCAA	AGGAAGTCCTCTGGCTCCTC	111
IL25	TGTGTCCACACTGTGTCAGC	GAAGACGGTCTGGTTGTGGT	89
IL4R	CAGAGCTGCCTGCTGTCAT	CTCTCCGGGATCTGAGGACT	80
IL13RA1	TCCCTCCAATTCCTGATCCT	TCCAGTGCAGGGTATCATCA	75
DCLK1	TAAGGCGCAGAGATACAGCA	GGTTCGGTAGAAGCTGCAAT	85
TSLP	ACTAAGGCTGCATTCGCACT	TTTTCCTCATTGCCTGGGTA	76
FFAR2	GCTTCGGGCCCTATAACATA	GCGTTGAGGGAGCTGAATAC	97
HDAC1	GGATCGGTTAGGTTGCTTCA	CCTCCCAGCATCAACATAGG	96
HDAC2	TGCAGTTCATGAAGACAGTGG	CACGCTATCCGTTTGTCTGA	87
HDAC3	GCTGCTGGACGTATGAGACA	GTCTGGATGGAGCGTGAAGT	110
HDAC6	CCCAAATCCATCGCAGATAC	GGCGAACGACTTAGAACTGG	86
HDAC9	GAACAGATGCGACAGCAAAA	CTTTTGTTGCCAAGGGAGAC	76
MCT1	CCGACTTCTGGCAAAAGAAC	GGCTTCTCAGCAGCGTCTAT	90
MUC1	GGATTTCTGAATTGTTTTTGCAG	ACTGTCTTGGAAGGCCAGAA	116
MUC2	GCACGTCTGCAACAAGGAC	CAAAGCCCTCCAGGCAGT	125

RETNLB	TCCCTCTGCTCCAAGAAAGA	CAAGCACAGCCAGTGACAAC	99
SLC2A5	GGTCATCTCCACCATCATCC	GCGCTCAGGTAGATCTGGTC	90
SLC5A1	TCTCATGAGCTCCCTGACCT	CTCTCTTCCGGATCTTGGTG	83
SLC5A8	TGGGACAAATTGGATGACAA	CCATCAGTGGAGTCCTTTCAA	86
TFF2	GCTGCTTCGACTCCCAAGT	CATGACGCACTCCTCAGACT	80
TFF3	TGTTCTGGCTGCTAGTGGTG	CAGTCCACCCTGTCCTTGG	112
IL6	TGGGTTCAATCAGGAGACCT	CAGCCTCGACATTTCCCTTA	116
IL18	CAATTGCATCAGCTTTGTGG	TCCAGGTCCTCATCGTTTTC	78
CXCL10	CCCACATGTTGAGATCATTGC	GCTTCTCTCTGTGTTCGAGGA	141
C3	ATCAAATCAGGCTCCGATGA	GGGCTTCTCTGCATTTGATG	76
CD14	GGGTTCCTGCTCAGATTCTG	CCCACGACACATTACGGAGT	164
CLDN3	ATCGGCAGCAGCATTATCAC	ACACTTTGCACTGCATCTGG	94
CTLA4	CTCCTGTACCCACCACCCTA	AGAATCTGGGCATGGTTCTG	84
DEFB1	TTCCTCCTCATGGTCCTGTT	CATCTTTGGAGCACACTTGC	114
OCLN	GACGAGCTGGAGGAAGACTG	GTACTCCTGCAGGCCACTGT	102
PLA2G4A	CGTACCCCTTGATCCTGAGA	CTTGGCCTTGCAGAAAAGTC	73
PTGES	TGTACGTAGTGGCCATCATCA	CTCCGTGTCTCTGAGCATCC	84
PTGS2	GAACTTACAGGAGAGAAGGAAATGG	TTTCTACCAGAAGGGCAGGA	94
SAA	GCTAAAGTGATCAGCGATGC	AGTGGTTGGGGTCCTTGC	145
**Housekeeping genes**			
GAPDH	ACCCAGAAGACTGTGGATGG	AAGCAGGGATGATGTTCTGG	79
RLP13A	ATTGTGGCCAAGCAGGTACT	AATTGCCAGAAATGTTGATGC	76
PPIA	CAAGACTGAGTGGTTGGATGG	TGTCCACAGTCAGCAATGGT	138

Supplementary Table 2.

Alpha diversity indices (Faiths PD) for BBE (left) and LB (right) groups for each segment. Pairwise Kruskal-Wallis. Dark grey: p > 0.1; Light grey: p 0.05 to 0.099; White: p< 0.05

BBE		
		**Faiths PD**
**JEJUNUM**	Crtl vs Od	0.834
	Crtl vs BBE	0.753
	Crtl vs BBE+Od	0.728
	Od vs BBE	0.834
	Od vs BBE+Od	0.165
	BBE vs BBE+Od	0.083
**ILEUM**	Crtl vs Od	0.668
	Crtl vs BBE	0.105
	Crtl vs BBE+Od	0.728
	Od vs BBE	0.197
	Od vs BBE+Od	0.519
	BBE vs BBE+Od	0.036
**CAECUM**	Crtl vs Od	0.439
	Crtl vs BBE	0.156
	Crtl vs BBE+Od	0.317
	Od vs BBE	0.529
	Od vs BBE+Od	0.817
	BBE vs BBE+Od	0.355
**PROX**	Crtl vs Od	0.908
	Crtl vs BBE	0.345
	Crtl vs BBE+Od	0.355
	Od vs BBE	0.563
	Od vs BBE+Od	0.406
	BBE vs BBE+Od	0.908
**DISTAL**	Crtl vs Od	0.046
	Crtl vs BBE	0.009
	Crtl vs BBE+Od	0.023
	Od vs BBE	0.208
	Od vs BBE+Od	0.752
	BBE vs BBE+Od	0.115

LB		
		**Faiths PD**
**JEJUNUM**	Crtl vs Od	0.916
	Crtl vs LB	0.208
	Crtl vs LB+Od	0.487
	Od vs LB	0.059
	Od vs LB+Od	0.298
	LB vs LB+Od	0.643
**ILEUM**	Crtl vs Od	0.568
	Crtl vs LB	0.654
	Crtl vs LB+Od	0.563
	Od vs LB	0.568
	Od vs LB+Od	0.606
	LB vs LB+Od	0.728
**CAECUM**	Crtl vs Od	0.606
	Crtl vs LB	0.199
	Crtl vs LB+Od	0.156
	Od vs LB	0.418
	Od vs LB+Od	0.401
	LB vs LB+Od	0.817
**PROX**	Crtl vs Od	0.728
	Crtl vs LB	0.172
	Crtl vs LB+Od	0.916
	Od vs LB	0.418
	Od vs LB+Od	0.728
	LB vs LB+Od	0.401
**DISTAL**	Crtl vs Od	0.046
	Crtl vs LB	0.005
	Crtl vs LB+Od	0.093
	Od vs LB	0.366
	Od vs LB+Od	0.834
	LB vs LB+Od	0.699

Supplementary Table 3.

Beta diversity (unweighted UniFrac) for BBE groups for each segment. Permanova (pairwise Kruskal-Wallis) for DMs in Figure 3. Dark grey: $p > 0.1$; Light grey: p 0.05 to 0.099; White: $p < 0.05$

BBE			
		Unweighted	
		p-value	q-value
JEJUNUM	Crtl vs BBE	0.290	0.290
	Crtl vs BBE+Od	0.080	0.134
	BBE vs BBE+Od	0.089	0.134
ILEUM	Crtl vs BBE	0.026	0.039
	Crtl vs BBE+Od	0.163	0.163
	BBE vs BBE+Od	0.026	0.039
CAECUM	Crtl vs BBE	0.032	0.096
	Crtl vs BBE+Od	0.103	0.155
	BBE vs BBE+Od	0.208	0.208
PROXIMAL	Crtl vs BBE	0.003	0.006
	Crtl vs BBE+Od	0.026	0.026
	BBE vs BBE+Od	0.004	0.006
DISTAL	Crtl vs BBE	0.005	0.008
	Crtl vs BBE+Od	0.014	0.014
	BBE vs BBE+Od	0.005	0.008

Supplementary Table 4.

Beta diversity (unweighted UniFrac) for LB groups for each segment. Permanova (pairwise Kruskal-Wallis) for DMs in Figure 3. Dark grey: p > 0.1; Light grey: p 0.05 to 0.099; White: p< 0.05

LB			
		Unweighted	
		p-value	q-value
JEJUNUM	Crtl vs LB	0.067	0.099
	Crtl vs LB+Od	0.022	0.066
	LB vs LB+Od	0.099	0.099
ILEUM	Crtl vs LB	0.356	0.356
	Crtl vs LB+Od	0.073	0.110
	LB vs LB+Od	0.030	0.090
CAECUM	Crtl vs LB	0.049	0.074
	Crtl vs LB+Od	0.030	0.074
	LB vs LB+Od	0.276	0.276
PROXIMAL	Crtl vs LB	0.006	0.018
	Crtl vs LB+Od	0.095	0.095
	LB vs LB+Od	0.030	0.045
DISTAL	Crtl vs LB	0.008	0.014
	Crtl vs LB+Od	0.009	0.014
	LB vs LB+Od	0.018	0.018

Supplementary Table 5. Relative expression and significance (*p*-value) of genes significantly influenced by diet, infection or interaction of both treatments. Significance determined as *p* ≤ 0.05. # indicates a trend of effect where *p* ≤ 0.1.

Statistical analysis was conducted separately for each probiotic treatment, using a GLM analysis comparing the effect of probiotic supplementation and infection (and their interaction) to the control-diet groups (no probiotics).

Immune function	Immune gene	Relative expression				Significance (*p*-value)					Significance (*p*-value)		
		Control	*O. dentatum*	BBE	*O. dentatum* + BBE	Diet	Infection	Interaction	LB	*O. dentatum* LB	Diet	Infection	Interaction
Th1	*IL1A*	5.3	4.7	7.4	6.5	0.048			5.9	5.6			
	IL1B	2.2	3.2	3.3	4.4	# 0.059	# 0.078		3.0	2.3			0.027
	IL8	7.7	5.5	11.0	8.7	0.007	# 0.054		9.4	7.9	# 0.098	# 0.082	
	IL12B	3.1	3.2	5.8	5.5	0.026			4.4	6.0	0.016		
	IFNG	3.9	3.1	5.4	4.0				6.4	5.2	0.002		
	TNF	8.9	29.6	19.1	18.6			0.014	26.9	31.2	# 0.091	0.026	
	TLR2	4.1	7.8	7.5	6.3			0.023	5.8	7.1		# 0.054	
	TLR3	1.8	3.0	2.3	2.0			0.047	2.2	2.6		0.005	# 0.054
	INOS	13.7	9.7	22.9	17.9	# 0.076			19.1	21.4	0.034		
	CCL3	2.5	3.8	3.7	3.1			# 0.07	5.6	4.4	0.012		# 0.096
	CXCL9	3.9	5.5	7.0	3.8			# 0.07	7.1	6.2			
Th2	*IL4*	44.5	93.3	40.5	88.7		0.003		51.2	105.9		0.013	
	IL13	5.5	35.4	9.6	19.0		0.005		5.5	38.9		0.001	
	ARG1	11.4	61.9	9.7	76.4		0.007		15.2	23.0			0.026
	CCL17	17.5	93.1	14.6	86.4		0.003		9.3	88.8		0.001	
	CCL26	2.2	9.3	3.1	3.9			0.033	2.7	6.6		0.001	
Treg	*TGFB1*	3.1	5.7	4.8	5.5		# 0.079		4.5	6.5		0.011	
Epithelial cell barrier and mucosal immune function	*IL4R*	5.7	17.1	9.4	6.7			0.001	8.7	10.8		0.032	
	DCLK1	12.1	49.3	23.2	16.7			0.009	25.1	31.1		0.019	
	TSLP	235.8	600.0	367.2	472.5		# 0.074		433.2	483.6		0.042	
	FFAR2	38.9	215.7	58.2	79.1			0.037	52.2	140.7		0.017	
	HDAC2	3.5	3.6	3.1	2.5	# 0.062			3.0	2.8	# 0.066		
	HDAC6	6.5	10.2	9.3	5.4			0.013	7.9	7.3			
	HDAC9	2.6	6.1	4.3	3.7			0.019	4.5	6.5		0.012	

	RETNLB	9.4	51.9	6.8	11.3	0.031	0.014		7.5	24.8		0.004	
Innate immune defence	*IL6*	4.6	15.9	5.1	8.9		0.02		6.2	8.9		0.04	
	C3	2.6	3.7	2.5	3.1		0.01		2.6	2.9		# 0.064	
	CD14	33.7	73.1	57.2	47.3			0.094	43.3	66.1			
	CTLA4	2.0	3.9	3.4	2.6			0.042	3.6	2.8			# 0.054
	CXCL10	3.8	4.0	6.7	5.3	0.04			6.4	4.7	# 0.059		
	PLA2G4A	4.2	5.1	5.7	3.7			0.036	4.8	4.5			
	PTGES	14.9	22.3	28.6	15.8			0.008	16.0	18.9			
	PTGS2	3.5	15.1	10.0	13.3		0.017		7.0	11.6		0.022	
